# Efficacy and Safety Evaluation of Mometasone Furoate in Treating Ocular Inflammation

**DOI:** 10.3390/pharmaceutics15010193

**Published:** 2023-01-05

**Authors:** Nayara Almeida Lage, Mayara Rodrigues Brandão de Paiva, Daniel Vitor Vasconcelos-Santos, Renes Resende Machado, Sílvia Ligório Fialho, Armando Silva-Cunha

**Affiliations:** 1 Faculty of Pharmacy, Federal University of Minas Gerais, Belo Horizonte 31270-901, Brazil; 2 Research and Development, Ezequiel Dias Foundation, Belo Horizonte 30510-010, Brazil; 3 Faculty of Medicine, Federal University of Minas Gerais, Belo Horizonte 30130-100, Brazil

**Keywords:** uveitis, mometasone, intravitreal administration, anti-inflammatory

## Abstract

Mometasone furoate (MF) is a medium-potency synthetic glucocorticosteroid with anti-inflammatory, antipruritic, and vasoconstrictive properties. However, its role in the treatment of ocular inflammation has not yet been explored. This work investigated the anti-inflammatory activity of MF in ocular tissues. First, the in vivo safety of the intravitreal (IVT) injection of MF (80, 160, and 240 µg) was evaluated via clinical examination (including the assessment of intraocular pressure), electroretinography (ERG), and histopathology. Second, MF was tested in an experimental model of bacillus Calmette–Guérin (BCG)-induced uveitis in Wistar rats. Intraocular inflammation was then evaluated via a slit-lamp and fundus examination, ERG, histopathology, and the quantification of pro-inflammatory markers. Intravitreal MF showed no toxicity in all the investigated doses, with 160 µg leading to attenuated disease progression and improvement in clinical, morphological, and functional parameters. There was a significant reduction in the levels of inflammatory markers (myeloperoxidase, interleukins 6 and 1β, CXCL-1, and tumor necrosis factor-alpha) when compared to the levels in untreated animals. Therefore, MF should be further investigated as a promising drug for the treatment of ocular inflammation.

## 1. Introduction

Inflammatory eye diseases are among the most prevalent causes of irreversible blindness worldwide [1]. Among them, uveitis is an important type, comprising a group of diseases characterized by primary inflammation of the uveal tract (composed of the iris, ciliary body, and choroid). Uveitis is associated with ocular complications such as cataracts, glaucoma, and retinal detachment, which can result in blindness [1,2].

The primary treatment of uveitis mainly consists of corticosteroids; however, their long-term use is associated with many local and systemic adverse effects [3,4]. In contrast, untreated/undertreated uveitis can lead to additional visual-threatening complications. Thus, prioritizing prompt control of intraocular inflammation and preventing ocular/systemic complications is the treatment paradigm. For this reason, the investigation of the biological effects of different/alternative steroids and their efficacy and safety for the treatment of intraocular inflammation is warranted [3,4,5].

The local administration of steroids, particularly through the intravitreal route, is being increasingly used for uveitis involving the posterior segment of the eye (intermediate, posterior, and panuveitis), especially in the setting of uveitic macular edema. Local steroids alone or in combination with other local or systemic anti-inflammatory drugs can be used to treat non-infectious uveitis, while reducing the burden of systemic steroids [5,6].

Mometasone furoate (MF) is a synthetic glucocorticosteroid of medium potency with anti-inflammatory, antipruritic, and vasoconstrictive properties that regulates the expression of numerous pro-inflammatory and anti-inflammatory genes. MF acts by preventing the influx of inflammatory cells into the nasal mucosa and inhibiting the expression of soluble mediators, such as histamine, interleukin (IL)-1, IL-4, IL-5, IL-6, IL-8, interferon-γ (IFN-γ), leukotrienes, and tumor necrosis factor-α (TNF-α) [7]. MF has been shown to be safe, effective, and even superior to other marketed products for controlling the inflammatory symptoms in allergic rhinitis [7,8]. However, at present, no study has investigated the ophthalmic application of MF. Therefore, this work aims to establish a safety profile for the IVT administration of MF and to investigate its anti-inflammatory effects in a rat model of BCG-induced uveitis.

## 2. Materials and Methods

### 2.1. Animals’ Preparation and Intravitreal Injections

Adult male Wistar rats (180–200 g) were used. The rats were kept under controlled conditions of temperature (27 ± 1 °C) and luminosity (12 h light/dark cycle). The animals received solid food and water *ad libitum*. All the experiments were conducted in accordance with the guidelines of the Association for Research in Vision and Ophthalmology (ARVO). The ethics protocol was approved by the Ethics Committee on the Use of Animals of Federal University of Minas Gerais (Comissão de Ética no Uso de Animais, CEUA-UFMG; approval number: 283/2018).

Before each IVT injection, electroretinogram, and/or slit-lamp analysis, animals were anesthetized via intraperitoneal injection of 90 mg kg^−1^ of ketamine (Ketamin; Dopalen, Ceva, Brazil) and 10 mg kg^−1^ of xylazine (Anasedan, Ceva, Brazil). The pupils were then dilated with 1% tropicamide drops (Mydriacyl; Alcon, Brazil) for 15 min, and the eyes were topically anesthetized with 0.1% phenylephrine/1% tetracaine eye drops (Anestésico, Allergan, Brazil). Once anesthetized and with dilated pupils, the IVT injections were administered using a 31-gauge needle (BD Ultrafine II, BD Consumer Healthcare, NJ, USA) with a 0.3-mL syringe. A volume of 5 μL of the MF suspension, dexamethasone (DX) suspension, or vehicle was slowly injected through the *pars plana* of both eyes, around 2 mm posterior to the limbus, directly into the vitreous. The needle remained in place for about 30 s to prevent reflux when it was removed. After the last experiment, all animals were euthanized with an intraperitoneal injection of 270 mg kg^−1^ of ketamine and 30 mg kg^−1^ of xylazine.

### 2.2. In Vivo Ocular Safety Evaluation

For evaluating the safety of IVT MF in adult male Wistar rats, animals were randomly divided into groups of six animals each, which received an IVT injection of the vehicle (sterile saline solution) or MF suspension (Supelco PHR1400, USA) at doses of 80 µg, 160 µg, and 240 µg in the right eye. After the administration of the IVT injections, the safety of MF was assessed via ophthalmic examination, intraocular pressure (IOP) measurement, electroretinographic examinations, and histopathological analyses. All the examinations were performed before and at 3, 7, and 15 days after the IVT injections.

IOP was monitored throughout the study using a veterinary tonometer (Tono-PenVet, Reichert, USA). These procedures were performed at baseline and 3, 7, and 15 days after the IVT injections under the same conditions and at the same time of the day to avoid circadian variation in IOP readings. To detect the signs of intraocular inflammation and/or infection, ophthalmic assessment included external eye examination, slip-lamp examination, and binocular indirect ophthalmoscopy (Eyetec OHD 4.2 ophthalmoscope, Eyetec Equipamentos Oftalmológicos, Brazil). Fundus images were registered with a smartphone camera coupled to the 90D lens.

Full-field electroretinography (ERG) was used to evaluate retinal electrical activity, indicative of the safety of IVT MF. ERG tests were performed in accordance with the International Society for Clinical Electrophysiology of Vision (ISCEV) guidelines [9]. ERG tests were performed at 3, 7, and 15 days after the IVT injection of MF (80, 160, and 240 μg/eye), or vehicle. As an internal control, we used the same eyes before the IVT injection. The ERG responses were acquired using a corneal bipolar contact lens electrode (Rat ERG Electrodes; LKC Technologies, USA). Two subcutaneous steel needle electrodes in the front (near the lateral side of both the eyes) were used as the reference and ground electrodes on the back of the animal. The impedance of the electrodes was evaluated before and after the examination and was less than 5 kX and 25 Hz. Light stimuli were provided using a Ganzfeld stimulator (ColorDome^TM^ desktop Ganzfeld, Diagnosys LLC, Littleton, MA, USA) placed inside a Faraday cage and controlled with a computerized system. Before ERG recordings, animals were previously adapted in the dark overnight. The scotopic ERG protocol recorded flashes of white light (6500 K) with a duration of 4 ms that were delivered in 11 steps of increasing luminosity (0.003–3 cd.m/s), having two main steps: a first at 0.01 cd.m/s, to evaluate the rods response, and a second at 3 cd.m/s, to analyze the mixed responses of cones and rods. Then, the rats were light-adapted with a background luminance of 3 cd.m/s for 10 min, followed by flashes of 3 cd.m/s for 4 ms and a 30 Hz white flicker stimulus of the same duration and luminance, to evaluate the cone response. Thereafter, ERGs responses were amplified and filtered between 0.3 to 300 Hz and analyzed using Espion E3 software (Diagnosys LLC, Lowell, MA, USA).

After ERG analysis, animals were euthanized via anesthetic overdose and the eyes were enucleated and fixed in Davidson’s solution (95% alcohol, formaldehyde, glacial acetic acid, and distilled water) over 24 h. The eyes were then dehydrated in a series of graded alcohol solutions, cleared with xylene, and embedded with paraffin. Paraffin blocks were cut into 5.0-µm-thick sections and stained with hematoxylin-eosin for the evaluation of alterations in the vitreal, retinal, and choroidal structures using a light microscope (Model Axio Imager M2, Zeiss, Oberkochen, Germany).

### 2.3. In Vivo Anti-Inflammatory Activity

#### 2.3.1. Induction of Uveitis and Treatment with MF

**Figure 1:**
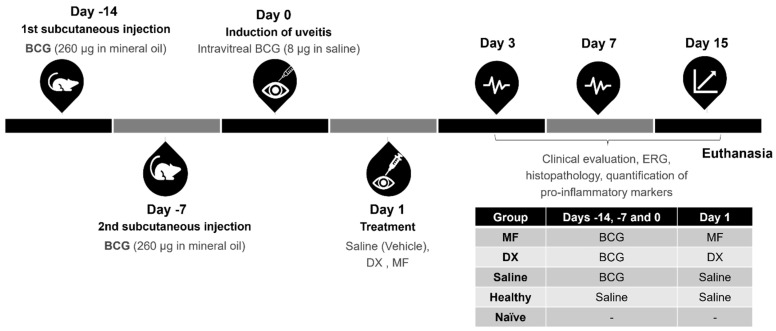
Flowchart of uveitis induction and intravitreal treatments. Animals were first pre-immunized with the subcutaneous injection of BCG on days −14 and −7, and uveitis was induced on day 0 with the intravitreal injection of BCG. On day 1, the animals were treated with saline, MF, or DX intravitreally. The healthy control group underwent IVT saline injection. BCG: bacillus Calmette–Guerin; MF: mometasone furoate; DX: dexamethasone; IVT: intravitreal.

Uveitis was induced in Wistar rats by using BCG (bacillus Calmette–Guérin), as previously described by Castro et al. and Paiva et al. [10,11]. Briefly, animals were pre-immunized with two subcutaneous injections (260 μg; 2.6 mg/mL) of BCG (ImmunoBCG 40 mg, equivalent to >2.0 106 CFU/mg, Fundação Ataulpho de Paiva, Brazil) suspended in 100 μL of Montanide ISA50 V2 (injectable mineral oil, Seppic, France) on the back of each animal with an interval of 1 week each (days −14 and −7). Then, rats were anesthetized as described above and uveitis was induced in both eyes via IVT injections (5 μL) of a BCG suspension in sterile saline (1.6 mg/mL). Animals were randomly divided into three groups (*n* = 9) and then treated with IVT injection (5 μL) of vehicle, dexamethasone suspension (DX 160 μg; Sigma Aldrich, St. Louis, MO, USA), or MF (160 μg). A healthy control group received all the treatments (two subcutaneous injections and one IVT injection) of saline at the same time, as shown in Figure 1. A group of naïve animals (*n* = 4) was not submitted to any intervention and was used as a control for the quantification of pro-inflammatory markers. 

#### 2.3.2. Ophthalmic Evaluation

To evaluate the treatment efficacy, clinical examination included external inspection, slip-lamp biomicroscopy (Apramed HS5, São Carlos, Brazil), and indirect binocular ophthalmoscopy (Eyetec OHD 4.2 ophthalmoscope, Brazil), employing a widefield noncontact lens (90D Volk Digital Wide Field, Volk, Germany). Ophthalmic examination was performed before and 3, 7, and 15 days after the IVT injection of BCG [10,11]. A masked ophthalmologist examined all animals and documented the observations. The following parameters were assessed: conjunctival hyperemia, corneal transparency, keratic precipitates, congestion of the iris vessels, anterior chamber cells, flare and fibrin, lens transparency, and inflammatory cells in the anterior vitreous [10,12,13]. 

#### 2.3.3. Electroretinography and Histopathological Analysis

Full-field electroretinographic examinations (*n* = 6) and histopathological analysis (*n* = 3) were performed at 3, 7, and 15 days after treatment, to evaluate disease progression and treatment effect on retinal function and morphology. ERG and histological protocols were carried out as described in the in vivo safety study. Signs of inflammation in the vitreous, retina, and choroid were assessed via light microscopy (*n* = 3) [14,15,16].

#### 2.3.4. Quantification of Pro-Inflammatory Markers

To investigate the effect of MF on production of pro-inflammatory markers, their quantification was carried out in the eyes of euthanized rats at 3, 7, and 15 days after the induction of intraocular inflammation by IVT injection of BCG. The quantification of pro-inflammatory markers in the ocular tissues was performed in 4 animals from each group after euthanasia at 3, 7, and 15 days after uveitis induction. The concentrations of TNF-α, IL-1β, IL-6, and CXCL-1 were assessed via ELISA following the instructions provided by the manufacturer (DuoSet kits, R&D Systems, Minneapolis, MN, USA). For this, the eyes were enucleated and posterior segment tissues (the sclera, choroid, and retina) were harvested, weighed, and homogenized in phosphate-buffered saline containing Tween-20 (0.05%), phenylmethylsulfonyl fluoride (0.1 mM), benzethonium chloride (0.1 mM), EDTA (10 mM), aprotinin A (2 μg/mL), and bovine serum albumin (0.5%), followed by centrifugation (10,000 rpm) for 15 min at 4 °C. Supernatant samples were stored at −70 °C until the analysis of cytokine concentrations. All samples were analyzed in duplicate, the absorbance was measured at 450 nm, and the results were expressed as pg/100 mg of the tissue.

The remaining pellets were used to characterize the inflammatory infiltrate, measuring the activity of N-acetylglucosaminidase (NAG) and myeloperoxidase (MPO) present in macrophages and neutrophils, respectively. The assays were carried out according to the method described by Paiva et al. [17]. For this, pellets were homogenized using a trisodium phosphate buffer solution (0.1 M NaCl, 0.02 M Na_3_PO_4_, and 0.015 M Na_2_EDTA; pH 4.7) and centrifuged at 4 °C for 10 min at 10,000 RPM. Supernatants were removed, pellets were resuspended in 0.2% NaCl solution and 1.6% NaCl plus 5% glucose, and samples were quickly homogenized and divided equally for the NAG and MPO assays. The resulting samples were centrifuged at 10,000 RPM for 10 min at 4 °C and the remaining supernatant was discarded.

For the MPO assay, half of the pellets resulting from the centrifugation of each sample was resuspended in 0.05 M Na_3_PO_4_ buffer (pH 5.4) containing 0.5% (*w*/*v*) of hexadecyltrimethylammonium bromide, followed by three freeze-thawing cycles with liquid nitrogen and centrifugation at 10,000 RPM for 15 min at 4 °C. The supernatant was seeded in triplicate. Then, the substrate 3,3′,5,5′-tetramethylbenzidine (TMB; 3.8 mg/mL diluted in dimethyl sulfoxide—DMSO) was added and the plate was incubated at 37 °C for 5 min. H_2_O_2_ (0.002%) was then added, and samples were again incubated at 37 °C for 5 min. After 10 min, the reaction was stopped by adding 100 μL of H_2_SO_4_ (1 M), and the MPO activity was measured via the absorbance reading at 450 nm. Results were expressed as the optical density (OD)/100 mg of tissue.

For the indirect quantification of the NAG activity (Assay Kit from Sigma Aldrich, St. Louis, MO, USA), pellets were homogenized with saline/Triton solution (Saline 0.9% and Triton x-100, 1%) and then centrifuged at 4 °C for 10 min at 10,000 RPM. The supernatant was collected and diluted in phosphate-citrate buffer (0.1 M citric acid and 0.1 M Na_2_HPO_4_) to proceed with the NAG assay. A total of 100 μL of each diluted sample was seeded in triplicate. The substrate p-nitrophenyl-N-acetyl-β-D-glucosaminidase (2.2 mM) was then added, diluted in phosphate-citrate buffer, and the plate was incubated for 5 min at 37 °C. After the reaction, 0.2 M glycine buffer was added. The absorbance reading was obtained at 405 nm. Results were expressed as the optical density (OD)/100 mg of the tissue.

#### 2.3.5. Statistical Analysis

Results were expressed as the mean ± standard deviation. Statistical parameters were analyzed through one-way analysis of variance (ANOVA) followed by the Tukey’s posttest to compare significance between the different groups. GraphPad Prism 6 software (GraphPad Software Inc., USA) was used for the analysis, and *p* < 0.05 was regarded as statistically significant.

## 3. Results

### 3.1. Safety Study of the Intraocular Administration of MF

Ophthalmic and ERG examinations, IOP monitoring, and histopathological analyses were performed to assess the in vivo ocular safety of the IVT injection of MF at 80, 160, and 240 μg in rats.

**Figure 2:**
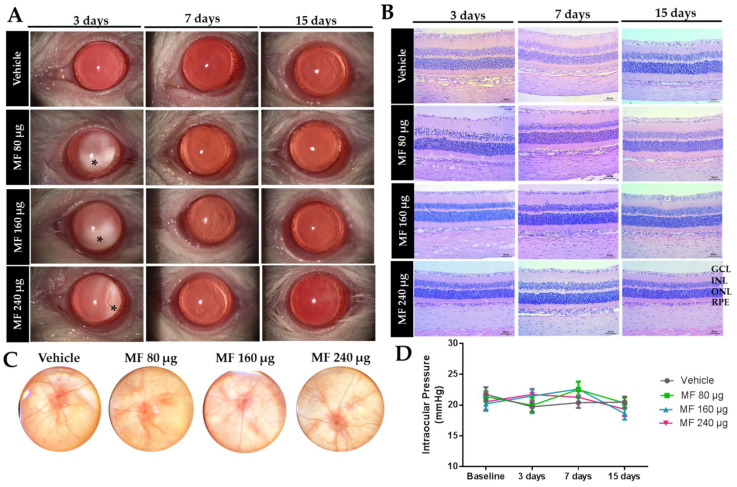
Mometasone furoate (MF) at doses of 80 µg, 160 µg, and 240 µg were shown to be safe for the IVT administration. (**A**) Representative slit-lamp photographs of the vehicle and MF-treated animals. (**B**) Light micrographs of the eyes after 3, 7, and 15 days of the IVT injection of the vehicle or MF (**C**) Photograph of indirect fundus ophthalmoscopy of the rats’ retina 15 days after the IVT injection. (**D**) The IOP of rats treated with the vehicle or MF at baseline, and 3, 7, and days after IVT injection. MF: mometasone furoate; GCL: ganglion cell layer; INL: inner nuclear layer; ONL: outer nuclear layer; RPE: retinal pigment epithelium cells; IVT: intravitreal; drug deposit in the vitreous (black asterisks). Bar = 50 μm.

Representative slit-lamp biomicroscopy images of the eyes 3 and 15 days after the IVT injection of MF and vehicle are shown in Figure 2A. In animals treated with MF, it was possible to visualize the drug in the vitreous 3 days after the administration. In all groups, the animals presented with iris congestion 3 days after the IVT injection, with subsequent regression over time. No other signs of inflammation/toxicity were observed in the period evaluated. This was in line with the results of the fundus examination, 15 days after the IVT administration of MF (Figure 2C), which showed no signs of retinal inflammation/toxicity, such as hemorrhage, vascular changes, or vitreous opacities; all the evaluated groups showed a normal optic disc. This was also confirmed via the histopathological analysis 3, 7, and 15 days after the IVT injection, represented by photomicrographs in Figure 2B. These show that the organization of the retinal layers was similar in each group (MF suspensions and vehicle), with no inflammatory cell infiltration, edema, or degeneration being observed. The IOP (Figure 2D) of animals treated with MF showed no significant differences when compared to those in the vehicle group, during the observation period of 15 days. These results suggest that IVT injections of 80, 160, and 240 μg of MF had no apparent toxicity to the retina, subsequently corroborating with clinical and electrophysiological results. 

**Figure 3:**
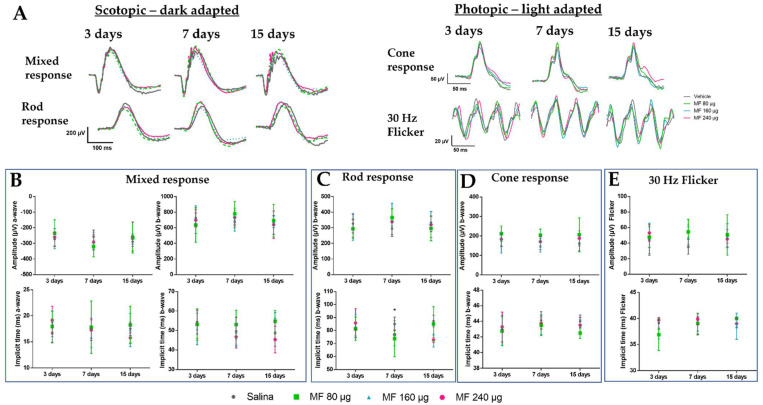
Electroretinography results in a safety study of the IVT injection of MF. (**A**) Graphs showing scotopic responses obtained from ERG at light intensities of 0.01 (rod response) and 3.0 cd.m/s (mixed response) and photopic responses obtained from ERG at light intensities of 3.0 cd.m/s (cone response) and flicker of animals receiving vehicle (gray line) and MF at 80 (green line), 160 (blue line), and 240 µg (pink line) IVT injection. (**B**–**E**) Mean and standard deviation of the amplitude and implicit time of *a* and *b* waves after 3, 7, and 15 days the IVT injection of the vehicle or MF (80, 160, and 240 µg. ERG in scotopic conditions (**B**) 0.01 cd.m/s and (**C**) 3.0 cd.m/s and in photopic conditions (**D**) 3.0 cd.m/s and (**E**) 30 Hz flicker (n = 6/group). * *p* < 0.05 vs. vehicle.

Records of electroretinographic examinations performed at 3, 7, and 15 days after the injection of the vehicle and MF at different concentrations are shown in Figure 3. The ERG findings indicate that there was no significant change in the retinal function that was suggestive of toxicity following MF or vehicle administration under the scotopic conditions at 0.01 cd.m/s (rod response; Figure 3C) and 3 cd.m/s(mixed response/combined rod-cone; Figure 3B), and in photopic conditions 3 cd.m/sand flicker (cone responses; Figure 3D,E, respectively). In addition, no notable changes were observed in the mean ERG curves (Figure 3A) throughout the observation period. However, animals treated with MF at 80 μg presented a significantly shorter implicit time for b-wave in the rod response 7 days after the IVT injection, when compared to the vehicle group (*p* < 0.05). This change was considered transient since this difference was not found 15 days after the IVT injection. Therefore, these findings support the safety of IVT MF in the proposed doses.

### 3.2. Treatment of BCG-Induced Uveitis with Intravitreal Mometasone

Rats with BCG-induced uveitis were treated with IVT MF, sterile saline (vehicle), or DX 1 day after disease induction. Intraocular inflammation was assessed and graded via clinical examination using slit lamp biomicroscopy, indirect binocular ophthalmoscopy, and clinical examination on 3, 7, and 15 days after uveitis induction.

All the animals in the BCG-induced uveitis group developed signs of intraocular inflammation in the anterior and posterior segment of the eye, and the healthy animals presented no abnormalities. After the IVT injection, vehicle-treated animals had more severe and prolonged signs of intraocular inflammation, with moderate cornea edema, severe iris and conjunctival hyperemia, inflammatory cells in the anterior chamber (AC) posterior synechia, and dense vitreous haze. In the IVT DX and MF-treated animals, these inflammatory signs were less significant and progressively improved, as animals presented with mild hyperemia of the iris and of conjunctiva and fewer AC cells. Of note, however, vitreous haze was observed in the IVT DX treated-group, but not in the IVT MF-treated group.

**Figure 4:**
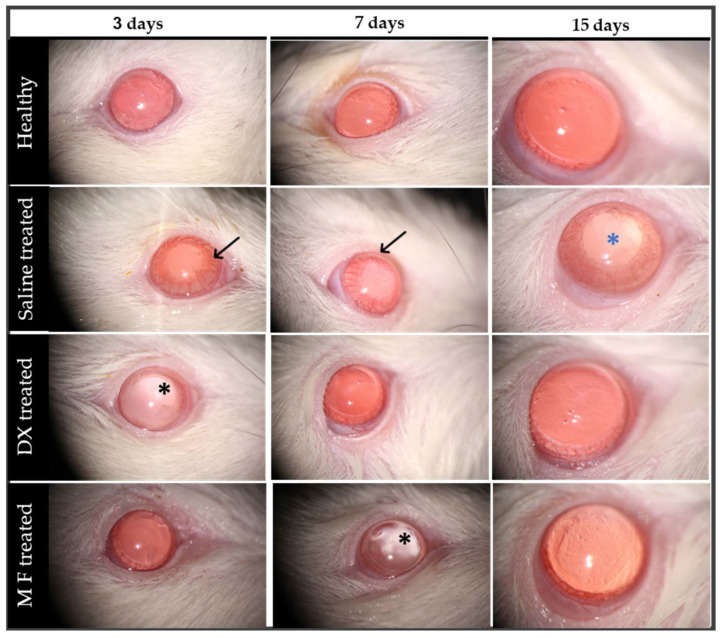
Representative images were obtained with a slit-lamp, 3, 7, and 15 days after rats were induced with BCG uveitis and treated with saline (vehicle), dexamethasone (DX), and mometasone furoate (MF). Clinical signs included iris congestion and posterior synechia (arrows), posterior subcapsular cataracts (blue asterisks), and it is also possible to visualize the drug deposit in the vitreous (black asterisks).

Figure 4 shows representative images from the external eye examination performed via slit-lamp biomicroscopy. We observed moderate iris hyperemia and posterior synechia in the saline-treated group. Furthermore, 15 days after uveitis induction, a posterior subcapsular cataract could also be seen. In the treated groups, it was possible to visualize the drug present into the vitreous after pupil dilation (DX after 3 days and MF after 7 days). Furthermore, in the DX group, mild hyperemia of the iris could be observed.

**Figure 5:**
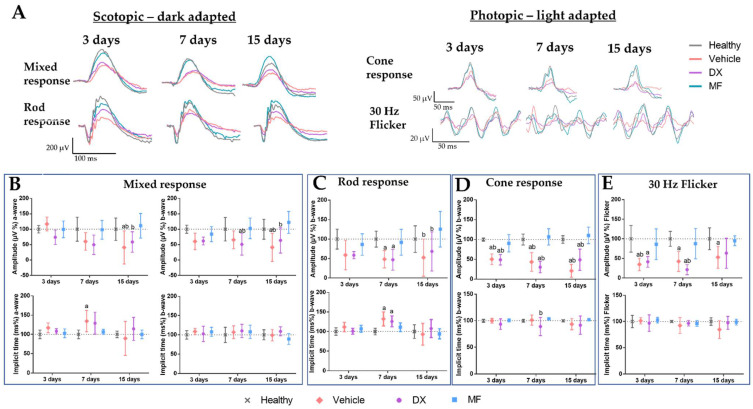
Electroretinography results in rats with BCG-induced uveitis showing improved responses after treatment with MF. (**A**) Graphs showing scotopic responses obtained from ERG at light intensities of 0.01 (rod response) and 3.0 cd.m/s (mixed response) and photopic responses obtained from ERG at light intensities of 3.0 cd.m/s (cone response) and flicker performed at 3, 7, and 15 days after the treatment of BCG-induced uveitis with the vehicle (pink line), DX (violet line), and MF (blue line), or in healthy animals (gray line). (**B**–**E**) The mean and standard deviation of the amplitude and implicit time of *a-* and *b*-waves in the scotopic condition (**B**) 0.01 cd.m/s and (**C**) 3.0 cd.m/s and in the photopic condition (**D**) 3.0 cd.m/s and (**E**) 30 Hz flicker (*n* = 6/group). ^a^ *p* < 0.05 vs. vehicle; ^b^ *p* < 0.05 vs. mometasone. MF: mometasone; DX: dexamethasone.

Representative ERG scotopic and photopic waveforms recorded 3, 7, and 15 days after the IVT injection of BCG are shown in Figure 5. As expected, there was a reduction in the retinal electrical activity after uveitis induction. ERG showed a decrease in the amplitude of *a-* and *b*-waves throughout the evaluation period, not only for animals treated with the vehicle but also for the animals treated with DX when compared to the healthy group. The amplitude of the cone responses was significantly reduced at all the time points in animals treated with the vehicle and in those treated with DX, when compared to the MF and healthy groups (Figure 5D). The amplitude of mixed responses was mainly reduced at 15 days (Figure 5B). For rod responses, there was a significant reduction in the amplitude in the DX group at 7 days, when compared to the vehicle group, and at 15 days when compared to the MF group (Figure 5C). Regarding the implicit time, there was a significant difference only in the groups treated with the vehicle and DX at 7 days; this difference was likely transient, not being observed at 15 days. 

**Figure 6:**
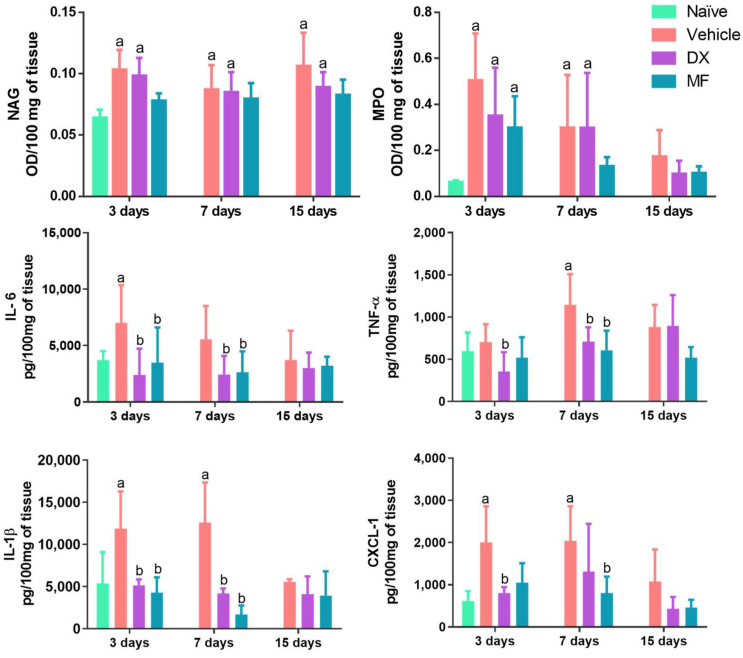
Effect induced by DX (violet bars), MF (blue bars), or vehicle (sterile saline; pink bars) on the concentrations of NAG, MPO, IL-6, TNF-α, IL-1β, and CXCL-1 in rats with BCG-induced uveitis. Concentrations were evaluated 3, 7, and 15 days after observing the BCG-induced uveitis. ^a^ *p* < 0.05 vs. naïve; ^b^ *p* < 0.05 vs. vehicle. MPO: myeloperoxidase; NAG: N-acetylglucosaminidase; IL: interleukin; TNF-α: tumor necrosis factor-alpha.

To characterize the effect of MF on intraocular inflammation, pro-inflammatory markers were quantified in the eyes of animals with BCG-induced uveitis and treated with vehicle (saline), MF, or DX, and in the eyes of healthy animals (naïve). As shown in Figure 6, increased concentrations of IL-6 (3 days), TNF-α (7 days), IL-1 β (3 and 7 days), and CXCL-1 (3 and 7 days) were observed in the vehicle-treated group when compared to the naïve group. A significant reduction in these biomarkers was observed in the eyes treated with DX or MF, when compared to the vehicle-treated group, mainly on day 7, with no significant difference between treatments. Finally, a general trend of reduction in the concentration of all biomarkers over time was noticed, mainly on day 15. The NAG and MPO levels were significantly higher in the uveitis group and in the DX group than in the naïve group. The NAG levels on days 3 and 7 were about 1.6 times higher in the DX and vehicle groups than in the naïve group, indicating heightened macrophage activation in these groups. In comparison, the MPO levels were about 8 and 4 times higher on days 3 and 7, respectively. The group treated with MF showed a significant increase in the MPO level on day 3, reducing by half on day 7. This suggests that IVT MF was able to modulate cytokines involved in the intraocular inflammatory response similar to DX.

**Figure 7:**
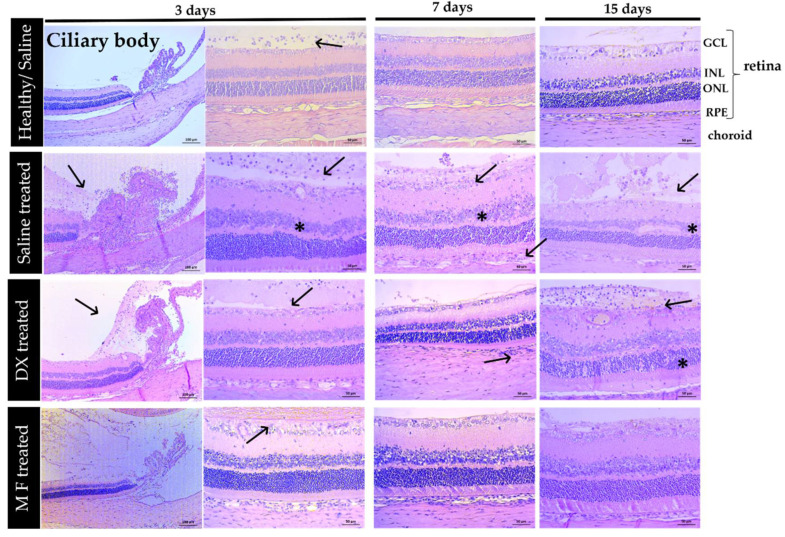
Representative histopathological sections of the main ocular structures affected by the inflammatory process (ciliary body, retina, and choroid) obtained 3, 7, and 15 days after uveitis induction, and subsequent treatment with the vehicle (saline), DX, or MF and healthy eyes with the IVT injection of saline. Black arrows indicate the presence of inflammatory cells; asterisks indicate cellular disorganization of the retinal INL and ONL. Abbreviations: GCL: ganglion cell layer; INL: inner nuclear layer; ONL: outer nuclear layer; RPE: retinal pigmented epithelium. Scale bar = 50 μm.

Histopathological evaluation was performed in healthy eyes receiving IVT saline and in eyes with BCG-induced uveitis treated with the vehicle (saline), MF, and DX at 3, 7, and 15 days after uveitis induction (Figure 7). In the saline group, mild infiltration of inflammatory cells was observed in the vitreous-retina interface on day 3, but the infiltration was reduced on day 7 or 15. As the only intervention in these animals was the subcutaneous and IVT injections of saline, this inflammatory infiltration was interpreted as a transient effect of the technique of the IVT injection. In animals with BCG-induced uveitis treated with a vehicle and DX, a strong inflammatory infiltrate was observed in the ciliary body, vitreous cavity, retina, and choroid at 3, 7, and 15 days, in addition to cellular disorganization of the retinal inner and outer nuclear layers. In the group treated with MF, we observed the presence of inflammatory cells at 3 days in the vitreous-retina interface. However, on days 7 and 15, the inflammatory infiltrate was reduced and there was no disorganization or thinning of the nuclear layers, in contrast to what was observed in animals treated with the vehicle or DX.

## 4. Discussion

We evaluated the safety of the IVT injection of MF with slit-lamp biomicroscopy, indirect ophthalmoscopy, fundus imaging, IOP measurement, retinal electrophysiology, and histopathology. In the current study, animals treated with MF (80, 160, and 240 µg) did not show any clinical, morphological, or electrophysiological evidence of adverse events during the 15 days of the study. Of note, histopathological evaluation did not reveal inflammatory cells, edema, or degeneration, and ERG did not reveal any changes in cone and rod responses during scotopic and photopic analyses in animals receiving IVT MF, when compared to those receiving saline. Taken together, these results fully support the safety of the studied doses of IVT MF in the retina of rats.

The intraocular anti-inflammatory effect of IVT MF was evaluated in an experimental model of BCG-induced uveitis in rats. The clinical signs of uveitis observed in this work are consistent with those obtained in previous studies using the same experimental model [10,12,18]. Compared to vehicle-treated eyes, MF reduced the clinical signs of inflammation on the 3rd day after uveitis induction, similar to DX-treated eyes.

MPO is a protein secreted by activated leukocytes, mainly found in the cytoplasmic granules of neutrophils, but also in monocytes. MPO is not found in healthy tissues; during the inflammatory process, there is vascular leakage and the recruitment of circulating neutrophils to the site of injury [11]. For this reason, it is considered an important biochemical marker to assess the degree of neutrophil infiltration in tissues. Otherwise, NAG is present in activated macrophages, either resident or infiltrated after inflammation, playing an important role in uveitis as an effector of innate and acquired immunity. NAG levels in naïve animals may be due to the presence of activated resident macrophages [11,17,19]. The reduction in the concentration of all biomarkers over time, mainly on day 15, is possibly due to their short half-life, as reported by Paiva et al., who showed high concentrations of IL-6 and TNF-α 2 days after uveitis induction with BCG, and a significant reduction from the 3rd to the 14th day [10].

The histopathological findings of uveitis induction were consistent with the quantification of intraocular inflammatory biomarkers. BCG-induced uveitis was characterized histologically by the infiltration of inflammatory cells and by high concentrations of pro-inflammatory cytokines, which may damage ocular tissues [20,21]. Both the quantification of inflammatory biomarkers and the histopathology findings indicate that MF was effective in reducing the concentration of pro-inflammatory cytokines and the recruitment of inflammatory cells in the current study.

Several studies have suggested that ERG may be a useful tool for monitoring and quantifying the severity of uveitis in experimental models. ERG parameters were significantly altered in animals with BCG-induced uveitis treated with saline or DX, indicating functional retinal damage [22]. The reduction in b-wave amplitude under photopic conditions and the abnormal oscillation of 30 Hz indicate an abnormal dysfunction of the post-receptor retinal structure (inner retina), and of the signal transmission from the photoreceptors (cones) to the bipolar cells. This may be caused by an increase in interneuronal distance secondary to edema in the context of inflammatory disruption of the blood-retinal barrier [23,24,25]. The increase in the implicit time, in turn, may indicate a delay in the response to the light stimulus, suggesting that the transmission of electrical signals from the photoreceptors to the bipolar and Müller cells was impaired [26]. The group treated with MF showed only isolated/mild ERG abnormalities, with ERG curves remaining similar to those of the naïve group. This indicates that IVT MF treatment may prevent ERG changes indicative of retinal dysfunction in the context of BCG-induced uveitis. 

## 5. Conclusions

The findings of the current study are the first to show the potential use of MF for treating ocular inflammation. It was demonstrated that MF may be safe for intravitreal administration in rat eyes. IVT MF showed a beneficial effect on the BCG-induced uveitis model in rats, alleviating intraocular inflammation and decreasing the expression of inflammatory markers. In addition, IVT MF preserved the retinal morphology and electrical function. Further studies are needed to better evaluate ocular anti-inflammatory properties of MF and their possible role as an alternative therapy for noninfectious uveitis.

## Data Availability

The data presented in this study are available on request from the corresponding author.
